# A Cross-sectional Study of the Impact of Blood Selenium on Blood and Urinary Arsenic Concentrations in Bangladesh

**DOI:** 10.1186/1476-069X-12-52

**Published:** 2013-07-01

**Authors:** Christine Marie George, Mary Gamble, Vesna Slavkovich, Diane Levy, Alauddin Ahmed, Habibul Ahsan, Joseph Graziano

**Affiliations:** 1 Department of International Health, Program in Global Disease Epidemiology and Control, Johns Hopkins Bloomberg School of Public Health, 615 N. Wolfe Street, Room W5535, Baltimore, MD E5535, USA; 2 Department of Environmental Health Sciences, Mailman School of Public Health, Columbia University, 722 W. 168th St, Rosenfield Bldg, New York 10032, USA; 3 Department of Environmental Health Sciences, Mailman School of Public Health, Columbia University, Trace Metals Core, New York 10032, USA; 4 Department of Biostatistics, Mailman School of Public Health, Columbia University, 722 W. 168th St, Rosenfield Bldg, New York, New York, USA; 5 University of Chicago Arsenic & Health Research in Bangladesh, Dhaka, Bangladesh; 6 Department of Health Studies, The University of Chicago Biological Sciences, 5841 S. Maryland Ave., MC 2007, Chicago, Illinois 60637, USA

**Keywords:** Selenium, Arsenic, Bangladesh, Pediatric populations

## Abstract

**Background:**

Arsenic can naturally occur in the groundwater without an anthropogenic source of contamination. In Bangladesh over 50 million people are exposed to naturally occurring arsenic concentrations exceeding the World Health Organization’s guideline of 10 μg/L. Selenium and arsenic have been shown to facilitate the excretion of each other in bile. Recent evidence suggests that selenium may play a role in arsenic elimination by forming a selenium-arsenic conjugate in the liver before excretion into the bile.

**Methods:**

A cross-sectional study of 1601 adults and 287 children was conducted to assess the relationship between blood selenium and urinary and blood arsenic in a study population residing in a moderately arsenic-contaminated rural area in Bangladesh.

**Results:**

The results of this study indicate a statistically significant inverse relationship between blood selenium and urinary arsenic concentrations in both adult and pediatric populations in rural Bangladesh after adjustment for age, sex, Body Mass Index, plasma folate and B12 (in children), and ever smoking and current betel nut use (in adults). In addition, there appears to be a statistically significant inverse relationship between blood selenium and blood arsenic in children.

**Conclusions:**

Our results suggest that selenium is inversely associated with biomarkers of arsenic burden in both adults and children. These findings support the hypothesis that Se facilitates the biliary elimination of As, possibly via the putative formation of a Se-As conjugate using a glutathione complex. However, laboratory based studies are needed to provide further evidence to elucidate the presence of Se-As conjugate and its role in arsenic elimination in humans.

## Background

Arsenic (As) can occur naturally in groundwater without an anthropogenic source of contamination. Groundwater pumped from approximately half of the roughly 10 million tubewells in Bangladesh do not meet the World Health Organization (WHO) guideline for As of 10 μg/L [1]. Many other countries around the world are also affected by elevated levels of As in drinking water including Chile, Mexico, Mongolia, Nepal, Vietnam, India, Taiwan, China, and the United States [2]. Exposure to elevated levels of inorganic As (As) is associated with cancers of the skin, bladder, and lung[3-5], developmental effects [6,7], cardiovascular disease [8,9], skin lesions [10,11], and decreased intellectual function in children [7,12,13].

In contrast, selenium (Se) is an essential mineral for human health which occurs in plants such as corn, wheat, and soybean. These selenocompounds include selenite, selenate, selenocystine, selenomethionine, selenohomocysteine, and Se-methylselenocysteine [14]. It is thought that selenate is reduced to selenite then to selenide by reduced glutathione [15]. Selenium is important for the reduction of hydrogen peroxide and damaging lipid and phospholipids into harmless products via the Se dependent enzyme, glutathione peroxidase (GPx) [16]. In addition to GPx other major groups of selenoproteins include include thioredoxin reductase, iodothyronine deiodinase,and selenoprotein P [17,18].

The maximum contaminant level (MCL) for Se specified by the Environmental Protection Agency is 0.05 mg/L [19]. Previous studies of individuals accidently ingesting toxic levels of Se containing products with a total dose ranging from 27 to 2387 mg had the following symptoms: nausea, vomiting, nail changes, hair loss, fatigue, breath smelling like garlic, diarrhea, and abdominal cramping [20]. In addition, in selenosis endemic areas of China studies have found disorders of the nervous system and paralysis [21,22].

The antagonistic relationship between As and Se was first report by Moxon in 1938 when he discovered that As protected against Se toxicity [23]. This effect has since been observed in rats, rabbits, dogs, swine, and cattle [24]. Studies have indicated that Se and As facilitate the excretion of each other in bile [25,26]. A metabolic link between arsenite and selenite has been identified in the bile of rabbits injected with Se and As [27]. In vitro evidence suggests the potential for the formation of this Se-As conjugate using glutathione (GS) complex (GS_2_AsSe) in the liver which is excreted into bile [26]. Biliary excretion of As by this pathway would likely reduce the amount of As excreted in urine. However the existence of this conjugate in humans remains unknown. In addition, a study of rat kidney cells found that As and Se are concentrated and precipitated in the lysosomes of renal cells, forming an insoluble selenide (As_2_Se) that is eventually eliminated in the urine [28][15]. This could be another potential As removal pathway.

A recent cross-sectional study in adults in Araihazar, Bangladesh found that plasma Se was inversely associated with urinary and blood As concentrations, and genomic DNA methylation [29]. Consistent with this finding a case-cohort study conducted at the same study site found that participants with higher blood Se concentrations had significantly lower urinary As concentrations. In addition, it was found that those with blood Se below average were consistently at a greater risk of skin lesions associated with their As exposure [30]. In contrast, a study in West Bengal found no difference in the blood Se concentration in those affected by skin lesion verses controls, however the As exposure of neither group was well characterized [31].

A recent animal study found that dietary organselenium was effective in preventing As from entering the peripheral tissue of mice that ingested arsenite in drinking water [32]. A second animal study found that co-administration of sodium arsenite and selenite had a protective effect on liver enzyme activity, thiobarbituric acid reactive substances levels, as well on glutathione peroxidase and glutathione-S-transferase in comparison to As treated animals [33]. These animal studies provide promising evidence for the role of Se in reducing As induced oxidative stress and the body burden of As.

A small clinical trial in Bangladesh has shown suggestive evidence that vitamin E and Se can slightly improve As induced skin lesions [2]. There has only been one small study published to date evaluating the impact of daily supplementation of Se on blood As concentrations in humans. However this study conducted in Inner Mongolia combined a Se supplementation trial with an “As free” drinking water intervention making it difficult to determine the actual effect of the supplementation component [34].

The current study is the first evaluation of the relationship between As and Se in a pediatric population. The primary objective of this study is to test the hypothesis that blood Se is inversely associated with blood and urinary As concentrations in children and adults living in an As contaminated area of Bangladesh.

## Methods

### Adult population

The data for the adult population in this study is from participants enrolled in the Health Effects of Arsenic Longitudinal Study (HEALS). HEALS is an ongoing prospective cohort study in Araihazar, Bangladesh of 20,000 men and women ages 18–65 years old that started in the year 2000. The study area of the cohort contains a wide range of well water As concentrations. Baseline health information was collected from study participants as well as whole blood, urine, well water samples, a semi quantitative food frequency questionnaire to measure dietary intakes, and an extensive physical exam. The cohort is followed up at roughly two year intervals. The primary objective of this study is to investigate the health effects of As exposure from drinking water on individual level exposures. The study area is located in Araihazar upazilla which is a subdistrict, one of 507 upazillas located in Bangladesh. Araihazar has an area of 183 square kilometers and contains 12 unions [35]. In 2006–7, roughly 8,000 additional participants were recruited into the HEALS study. For the current study, we included a random sample of 1601 of these additional participants for analysis, using a random number generator. The average duration of use of wells for HEALS cohort participants is 10 and 8.3 years for males and females, respectively.

### Pediatric population

The pediatric population in this study are children 8–11 years old living in the HEALS cohort study area who were enrolled in a cross-sectional study designed to assess the relationship between As and manganese(Mn) exposure and cognitive and motor function in children. This study recruited 301 children in the following four group based on their well-water concentrations: high As-high Mn (As > 10 μg/L and Mn > 500 μg/L); high As-low Mn; low As-high Mn; and low As-low Mn. Children were recruited between January and December 2008. Details on complete enrollment procedures have been previously published [7]. Only 287 of these children had blood Se information available, and thus included in the present study.

### Laboratory methods

Blood samples were collected by venipuncture in Ethyl-enediaminetertraacetic acid (EDTA) containing Vacutainers tubes. These were placed in cool packs which were designed to keep blood samples at 0 degrees Celsius. Samples were transported within 6 hours to our local laboratory in Araihazar, Bangladesh to be stored at −80 Celsius. Samples were then shipped on dry ice to Columbia University for analysis. Urine samples were collected in 50 ml acid washed polypropylene tubes, and kept in portable coolers. Within 6 hours all urine samples were frozen at −20 degrees Celsius, and then shipped to Columbia University on dry ice.

#### Blood arsenic and selenium

Whole blood samples were analyzed for As and Se concentrations using a Perkin-Elmer Elan DRC II Inductively Coupled Plasma Mass Spectrometry (ICP-MS) equipped with AS 93+ autosampler. Matrix induced interferences were corrected for using internal standards. Whole blood samples were thawed and diluted with 1%HNO_3_ + 1% Methanol + 0.2% Triton X-100 + 0.5% NH_4_OH. Samples were then centrifuged for 10 minutes at 3500 rpm [36]. Our laboratory participates in a quality control program for blood As, and Se which is coordinated by the Institut de Santé Publique du Québec (Québec, Canada).

#### Plasma folate and vitamin B-12

Plasma folate and total cobalamin were measured by radioimmunoassay using the Quantaphase II radioimmunassay (*Rad Laboratories*, Richmond, CA). This method is performed by heating the sample to 100 degrees Celsius to denature endogenous binding substances. For the determination of folate, pteroylgutamic acid was used for calibration, and ^125^I-labeled analog was used as the tracer. For the determination of cobalamin, cyanocobalamin was used for calibration, and ^57^Co-labeled analogy was used as the tracer [37].

#### Total urinary arsenic

Total urinary As was measured using a Perkin-Elmer AAnalyst 600 graphite furnace system, and adjusted for urinary creatinine (Cr) concentrations according to pub-lished methods [38]. Our laboratory participates in a quality control program for total urinary As which is coordinated by the Institut de Santé Publique du Québec (Québec, Canada).

#### Water arsenic

Water As samples were collected in 20 ml acid-washed tubes and As concentrations were measured using Inductively Coupled Plasma-Mass Spectrometry with a detection limit of 0.1 μg/L at the Geochemistry Research Laboratory at Lamont Doherty Observatory (LDEO) at Columbia University [39].

### Statistical analysis

The primary hypothesis of this study is that As exposure is inversely associated with Se status. The primary outcome variables in this study are blood and urinary As concentrations. The main predictor of interest is blood Se, and study covariates are age, sex, BMI, plasma folate and B12 (in children), ever smoking (for adults), current betel nut use (for adults), and urinary creatinine. We selected these covariates because they are known to influence As metabolism [29,40,41]. Plasma folate and B12 data were collected as part of the larger study of As and manganese exposure and cognitive and motor function in children. Unfortunately, plasma was not available for adults enrolled in HEALS study therefore we lack data on plasma folate and B12 in the adult population.

Descriptive statistics were calculated for the general characteristics of the study population, and for relevant micronutrients and urinary and blood As concentrations. We examined bivariate associations between micronutrients in blood, blood and urinary As, and population characteristics using Spearman correlation coefficients.

Blood and urinary As and blood Se were treated as continuous variables. Linear regression was used to examine the association between outcome variables and blood Se. All variables that were not normally distributed were log transformed. Blood Se values were also divided into five categories and covariate adjusted means were calculated. All analyses were performed using SAS, version 9.3 (SAS Institute Inc., Cary, NC, USA).

### Ethical approval and Consent

Written Informed consent and child assent were obtained from study participants by trained field physicians. This study was approved by the institutional review boards of Columbia Presbyterian Medical Center and the Bangladesh Medical Research Council.

## Results

### Adult population

The general characteristics of the 1601 adult participants are outlined in Table 1. The water As exposure in this population was low to moderate. The mean water As concentration was 52 μg/L. The mean age was 37 years and 62% of the study population was female. The average BMI was 19.7. Betel nut was currently used by 29% of the study population, and current cigarette use was 7%. Household landownership was 47%. The blood and urinary As concentrations in the population was 7 μg/L and 90 μg/L, respectively. Using Spearman correlation coefficients, blood Se was significantly associated with urinary creatinine (r = 0.09, p < 0.05), urinary As per gram creatinine (r = −0.08, p < 0.05), age (r = −0.10, p < 0.0001), and BMI (r = 0.10, p < 0.0001) (Table 2).

**Table 1:** **General characteristics of the study population **^**a**^

	**Mean±SD (range) or percent**		
Variable	Adult Population (n=1601)	Pediatric Population (n=287)	P-value^b^
Age (Years)	37 ± 11 (18–60)	9.64 ± 0.78 (8–11)	<.0001
BMI	19.7 ± 3.1 (10.1-34.2)	14.1± 1.33 (11.46 - 25.1)	<.0001
Male	38%	50%	0.0003
Household Land Ownership	47%	54%	0.03
Current Betel Nut Use	29%	N/A	N/A
Current Cigarette Use	7%	N/A	N/A
B-As (μg/L)	7 ± 5.85 (0.57 - 56.3)	4.80 ±3.21 (0.873-21.0)	<.0001
U-As (μg/L)	90.7 ± 104 (1–1138)	78.2 ± 72.5 (6–461)	0.01
Urinary Creatinine (mg/dL)	48 ± 38.3 (3.7 - 338.8)	35.0 ± 24.2 (4.9-144.9)	<.0001
Water As (μg/L)	52.0 ± 73.0 (0.1 - 700.0)	43.6 ± 74.0 (3.0 - 464.0)	0.0702
Urinary As per gram Creatinine (μgAs/g Cre)	226 ± 257 (10.0 - 4135)	247 ± 185 (29.8 - 1265)	0.1072
Blood Se (μg/L)	127 ± 20.5 (59.5-222)	105±17.2 (64.1-174)	<.0001
Baseline Plasma Folate (nmol/L)	N/A	13.0 ± 5.37 (4.05 - 32.9)	N/A
Baseline Plasma B12 (nmol/L)	N/A	279.0 ± 142 (34.5 - 819)	N/A

^a^ Urinary Arsenic (U-As) and Blood Arsenic (B-As) ^b^ P-values were calculated using a chi-square test for categorical variables and a 2 sample t-test for continuous variables.

**Table 2:** **Spearman correlation coefficients for blood Se versus outcome and demographic variables **^**a**^

	**Blood Se**			
	Adult Population	P-value	Child Population	P-value
U-As	0.006	0.82	−0.1	0.10
Urinary Creatinine	0.09	0.0006	0.05	0.37
Urinary As per gram Cr	−0.08	0.001	−0.20	0.001
B-As	0.03	0.31	−0.13	0.02
Plasma Folate	N/A	N/A	−0.09	0.12
Plasma B12	N/A	N/A	0.18	0.002
Age	−0.10	<0.0001	−0.13	0.02
BMI	0.10	<0.0001	0.09	0.14
Sex	0.12	<0.0001	0.04	0.65

a. Urinary Arsenic (U-As) and Blood Arsenic (B-As).

In the linear regression model presented in Table 3, the parameter estimates are the change in the mean concentrations of the outcome variables associated with a one unit increase in blood Se with and without adjusting for sex, BMI, current betel nut use, and urinary creatinine (when applicable). A statistically significant inverse association was found between blood Se concentrations and total urinary As concentrations in both the unadjusted and adjusted models. However, there was not a statistically significant association found between blood Se and As.

**Table 3:** **Estimated parameters and 95% confidence intervals for the association between blood Se (μg/L) (continuous variable) and urinary and blood As concentrations **^**a**^

**Outcome variables**	**(95% CI)**		**(95% CI)**	
**Adult Population (n=1601)**	**Unadjusted covariates**	**p-value**	**Adjusted covariates**^**b**^	**p-value**
Log Total	−0.003 (−0.005, -0.0009)	0.005	−0.002 (−0.004, -0.00003)	0.047
U-As (μg/L)				
Log Total	0.001(−0.0007, 0.003)	0.25	0.001 (−0.0007, 0.003)	0.24
B-As (μg/L)				
**Pedatric Population (n=287)**	**Unadjusted covariates**	**p-value**	**Adjusted covariates**^**c**^	**p-value**
Log Total	−0.006 (−0.01, -0.002)	0.006	−0.006 (−0.01, -0.001)	0.01
U-As (μg/L)				
Log Total	−0.004 (−0.008, -0.00002)	0.002	−0.004 (−0.009, -0.0001)	0.04
B-As(μg/L)				

^a^ Blood Arsenic (B-As) and Urinary Arsenic (U-As) ^b^ Adjusted for age, sex, BMI, current betel nut use, and urinary creatinine (log) for the outcome of urinary arsenic ^c^ Adjusted for sex, BMI, plasma folate (log), plasma B12 (log), and urinary creatinine (log) for the outcome of urinary arsenic.

Adjusted mean values of total blood and urinary As by blood Se quintile are presented in Table 4. Decreasing urinary As concentrations were observed with increasing quintiles of blood Se with a threshold effect for both outcome variables after the second quintile. There was no trend observed for blood As with increasing quintiles of blood Se.

**Table 4:** **Adjusted mean (95% CI) of outcome variables by blood Se quintile in the adult population(n=1601) **^**a**^

	**Se category [μg/L; mean(range)]**^**a**^				
	1	2	3	4	5
Adult Population	59.5 - 109.6 (n=320)	109.7-120.3 (n=320)	120.4-130.2 (n=321)	130.3-143.7 (n=319)	143.8-221.97 (n=321)
Total U-As (μg/L)	63.7(58.3-68.9)	52.1(47.7-56.8)	56.4(51.7-61.5)	58.2(53.3-63.5)	53.0 (48.5-57.9)
Total B-As (μg/L)	5.5(5.1-6.0)	5.0 (4.6-5.4)	5.4 (5.0-5.8)	5.5 (5.1-5.9)	5.5(5.1-5.9)

^a^ These are geometric means. B-As is for blood arsenic and U-As is for urinary arsenic. Adjusted for age, sex, BMI, ever smoking, and current betel nut, and urinary creatinine (log) for the outcome of urinary arsenic.

### Child baseline data

Population characteristics for the 287 child study participants are presented in Table 1. The water As exposure in this population was also low to moderate. The mean water As concentration was 43.6 μg/L. Fifty percent of participants were female and household landownership was 54%. The mean age of the children enrolled in the study was 9.64 years, and the mean BMI was 14.1. The mean blood and urinary As concentrations were 4.8 μg/L and 78 μg/L, respectively. Using Spearman correlation coefficients, blood Se was significantly associated with urinary As per gram Cr (r =−0.20, p < 0.05), blood As (r = −0.13, p<0.05), plasma B12 (r = 0.18, p < 0.05), and age (r = −0.13, p<0.05) (Table 2).

The parameter estimates in Table 3 for the linear regression models represent the change in the mean concentration of the outcome variables associated with a one unit increase in blood Se with and without adjusting for sex, BMI, plasma folate (log), plasma B12 (log), and urinary Cr (when applicable). A statistically significant inverse association was found between blood Se and urinary and blood As in the adjusted and unadjusted models.

The adjusted mean values of total blood and urinary As by Se quintile are presented in Table 5. In the pediatric population, decreasing urinary and blood As concentrations were observed with increasing quintiles of blood Se with the suggestion of a threshold effect for both outcome variables after the second quintile.

**Table 5:** **Adjusted mean (95% CI) of outcome variables by blood Se quintile (n=287) in child population **^**a**^

	**Se category [μg/L; mean(range)]**^**a**^				
	1	2	3	4	5
Pediatric Population	64.1 - 90.4	90.5-99.4	99.5-109.6	109.6-119.6	119.7-173.6
	(n=55)	(n=60)	(n=56)	(n=57)	(n=59)
Total U-As (μg/L)	65.8 (55.2, 78.3)	60.8 (51.5, 71.8)	52.5 (44.2, 62.3)	48.6 (41.0, 57.7)	50.7 (42.8, 60.0)
Total B-As (μg/L)	4.6 (3.9, 5.4)	4.2 (3.6, 4.9)	3.8 (3.2, 4.4)	3.6 (3.1, 4.2)	3.7 (3.2, 4.4)

^a^ These are geometric means. B-As is for blood arsenic and U-As is for urinary arsenic. Adjusted for sex, BMI, plasma folate (log), plasma B12(log), and urinary creatinine (log) for the outcome of urinary arsenic.

## Discussion

To our knowledge, this is the first study evaluating the relationship between As and blood Se in children. In this cross-sectional study of 1601 adults and 287 children we found that blood Se is inversely associated with urinary As concentrations after adjustment for age, sex, BMI, plasma folate and B12 (in children), and ever smoking and current betel nut use (in adults). In addition, blood Se and As were found to be inversely associated in the pediatric population. These findings are consistent with our original study hypothesis.

The statistically significant association found between blood Se and urinary and blood As are intriguing given that these study populations have low to moderate As exposure. In Pilsner et al., where a statistically significant association was found between both urinary and blood As and plasma Se, the mean water As exposure was 113.6 μg/L [29]. This was more than twice that in our present study. Chen et al. also found a statistically significant association between urinary As and blood Se As at mean water As concentrations of 101.8 μg/L [30]. This finding suggests that even at As concentrations lower than previously reported there exists an inverse relationship between As and Se.

Urinary creatinine which is influenced by the dietary intake of creatine and muscle mass was significantly lower in children versus adults (p-value < .0001). This is consistent with previous evaluations in the study area [29,42]. Urinary As was also found to be significantly lower in the pediatric population (p-value < .01). However after adjustment for hydration status using urinary creatinine there was no statistically significant difference observed in urinary As concentrations between the two study populations.

The blood Se concentrations were found to be significantly lower in the pediatric versus adult population (p-value < .0001). This is consistent with previous studies that have observed increasing Se status with age [43-45]. BMI was found to be positively associated with blood Se in children. This finding is likely attributable to a better overall diet in those children with a higher BMI.

The observed blood Se concentrations (59.5-222 μg/L) in the present study are not particularly low when compared to values globally which range from a mean blood Se of 62 μg/L in Finland to 229 μg/L reported in the central states of the United States [46]. Previous studies of children have reported mean values of blood Se ranging from 42 μg/L to 120 μg/L [47]. Thus, this region of Bangladesh does not appear to be associated with Se deficiency.

There is a growing body of scientific literature suggesting a potential elimination route of As through the formation of Se-As conjugate(GS_2_AsSe) in the liver which is excreted into bile [28,48]. This conjugate thus far has only been identified in animals models [27,49]. In our current evaluation of an adult population, we observed a statistically significant association between blood Se and total urinary As, however no such association was found for blood As. This result is consistent with findings in Chen et al. [30]. In contrast, Pilsner et al. found a statistically significant association between Se status and total blood and urinary As concentrations in an adult population, however plasma rather than whole blood Se was used as the marker of nutritional status [29]. In our pediatric population, we observed a statistically significant inverse association between blood Se and both total urinary and blood As. The reason for the statistically significant association between blood Se and As observed in children but not adults is unclear and warrants future investigation.

For the adult population the greatest reduction in mean adjusted urinary As concentrations occurred from the first to the second quintile, while for the pediatric population this occurred from the second to the third quintile. The blood Se cutoff for both was at approximately 110 μg/L. These finding suggest a threshold effect in both adult and pediatric populations after which the blood Se concentration has little effect on urinary As concentrations. A similar threshold effect was observed in Pilsner et al. [29].

A limitation of this study was that we did not measure urinary metabolites of As in our adult and pediatric study population. Pilsner et al. found that a higher proportion of dimethylarsinic acid (DMA(III) and DMA(V)), was positively associated with blood Se [29]. This suggests the possibility that higher blood Se is associated with increased inorganic As metabolism.

## Conclusions

The results of this study indicate a statistically significant inverse relationship between blood Se and urinary As concentrations in both adult and pediatric populations in rural Bangladesh. In addition, there appears to be a statistically significant inverse relationship between blood Se and As in children. These findings suggest the potential for the hypothesized elimination mechanism for As, possibly via the the Se-As conjugate (GS_2_AsSe). However, because we conducted a cross-sectional analysis we cannot determine if this relationship is causal. Laboratory based studies are needed to elucidate if the Se-As conjugate (GS_2_AsSe) is an important route of As elimination in humans. Further research is needed to investigate nutritional influences of Se on As metabolism in pediatric populations.

## Abbreviations

As: Arsenic; BMI: Body Mass Index; Cr: Urinary creatinine; GS: Glutathione; GPx: Glutathione peroxidase; HEALS: Health Effects of Arsenic Longitudinal Study; ICP-MS: Inductively Coupled Plasma Mass Spectrometry; Mn: Manganese; Se: Selenium; WHO: World Health Organization.

## Competing interests

The authors declare that they have no competing interests.

## Authors’ contributions

All authors have read the final version of the manuscript and have given final approval of this version of the manuscript to be published. CMG conducted the analysis of study data and completed the writing of this manuscript. VS was responsible for the chemical analysis. MVG planned the study of the pediatric population and reviewed the manuscript. DL managed the acquisition of the data and reviewed the manuscript. AA oversaw the activities of the field team, and reviewed the finalized manuscript. HA designed the study of the adult population and reviewed the finalized manuscript. JG designed the study of the adult population, assisted in the analysis of the study data, and critically reviewed the manuscript.
